# Impact of Requiring Influenza Vaccination for Children in Licensed Child Care or Preschool Programs — Connecticut, 2012–13 Influenza Season

**Published:** 2014-03-07

**Authors:** James L. Hadler, Kimberly Yousey-Hindes, Kathy Kudish, Erin D. Kennedy, Vincent Sacco, Matthew L. Cartter

**Affiliations:** 1 Connecticut Emerging Infections Program, Yale School of Public Health, New Haven, Connecticut; 2 Connecticut Dept of Public Health; 3 Division of Immunization Services, National Center for Immunization and Respiratory Diseases, CDC

Preschool-aged children are at increased risk for severe influenza-related illness and complications. Congregate child care settings facilitate influenza transmission among susceptible children. To protect against influenza transmission in these settings, in September 2010, Connecticut became the second U.S. state (after New Jersey) to implement regulations requiring that all children aged 6–59 months receive at least 1 dose of influenza vaccine each year to attend a licensed child care program. To evaluate the impact of this regulation on vaccination levels and influenza-associated hospitalizations during the 2012–13 influenza season, vaccination data from U.S. and Connecticut surveys and the Emerging Infections Program (EIP) were analyzed. After the regulation took effect, vaccination rates among Connecticut children aged 6–59 months increased from 67.8% during the 2009–10 influenza season to 84.1% during the 2012–13 season. During the 2012–13 influenza season, among all 11 EIP surveillance sites, Connecticut had the greatest percentage decrease (12%) in the influenza-associated hospitalization rate from 2007–08 among children aged ≤4 years. Additionally, the ratio of the influenza-associated hospitalization rates among children aged ≤4 years to the overall population rate (0.53) was lower than for any other EIP site. Requiring vaccination for child care admission might have helped to increase vaccination rates in Connecticut and reduced serious morbidity from influenza.

The Advisory Committee on Immunization Practices first recommended annual influenza vaccination for children aged 6–23 months in 2004 (1) and for children aged 24–59 months in 2006 (2). In 2012–13, the national vaccination level among children aged 6–59 months was 69.8%, the lowest among vaccines routinely recommended for this age group except for rotavirus and hepatitis A vaccines (3,4). In the United States, 60% of preschool-aged children receive nonparental care each week, and among these children, 60% receive center-based care, which includes child care centers, preschools, and pre-kindergarten programs (5). To protect against outbreaks of disease, all 50 states have legal requirements for specific immunizations for children attending these centers (6). However, only two states, Connecticut and New Jersey, require immunization against influenza. In January 2014, New York City became the first reported municipality to pass a similar requirement.

Connecticut first required that all children aged 6–59 months in licensed child care receive at least 1 dose of influenza vaccine by January 1 of each year, beginning September 2010. One year later, the same requirement was made for all children aged 24–59 months who were enrolled in a preschool program.

Data from multiple surveys were used to estimate vaccination rates among children aged 6–59 months in Connecticut. Estimates of influenza vaccination coverage from the National 2009 H1N1 Flu Survey and Behavioral Risk Factor Surveillance System (BRFSS) for the 2009–10 influenza season (7) and from the National Immunization Survey and BRFSS for the 2012–13 influenza season (3) were used to compare Connecticut’s and national influenza vaccination rates before and after implementation of the Connecticut requirement. Data from a survey of all licensed child care facilities in Connecticut conducted in March 2013 were used to determine vaccination levels among child care attendees (but not among preschoolers).

Surveillance data from EIP were used to examine changes in influenza-associated hospitalization rates in Connecticut,* compared with other EIP sites before and after implementation of the Connecticut influenza vaccination requirements. Because different influenza strains tend to affect different age distributions, data from 2007–08 were chosen *a priori* as the comparison with the post-requirement data (2012–13) to compare the two most recent influenza seasons during which the same influenza subtype (influenza A [H3N2]) was the predominant circulating strain. Eleven EIP sites, including two in New York, have been conducting active population-based surveillance for laboratory-confirmed influenza-associated hospitalizations for all ages since the 2005–06 season.

During 2009–10, the season before the state’s influenza vaccination requirement took effect, 67.8% (95% confidence interval [CI] = 61.1%–74.5%) of Connecticut children aged 6–59 months received a vaccination for seasonal influenza (Figure).† During the 2012–13 season, the seasonal influenza vaccination rate increased to 84.1% (CI = 78.2%–90.0%). The increase of 16.3 percentage points in Connecticut was greater than the national increase of 11.9 percentage points (from 57.9% to 69.8%), comparing the same age group for the same two seasons, but the difference is not statistically significant (Figure).

Among 11 EIP sites during the 2007–08 influenza season, Connecticut ranked third-highest in incidence of influenza-associated hospitalizations among children aged ≤4 years (58.6 per 100,000). During the 2012–13 season, Connecticut dropped to seventh (51.5 per 100,000) and was one of only two sites to record a decrease in incidence (12%) among children aged ≤4 years (Table 1).

During the 2007–08 influenza season in Connecticut, the ratio of the rate of influenza-associated hospitalization among children aged ≤4 years to the rate overall (i.e., for all ages) was 1.18 (Table 2). For the 2012–13 influenza season, the Connecticut ratio was 0.53, lower than any other EIP site.

Results from the Connecticut child care survey indicated that, as of December 31, 2012, licensed child care enrollment included 55,640 children who were aged 6–59 months. Of these, 87.1% had received ≥1 dose of influenza vaccine for the 2012–13 season. In total, 5.1% of children enrolled in the survey were listed as exempt from influenza vaccination for either religious or medical reasons, compared with 1.7% for all other vaccinations.

## Editorial Note

Requirements for vaccination against communicable diseases in licensed child care have long been important as a component of disease control in congregate care settings. Children aged ≤4 years are at greater risk for severe complications from influenza than older children (1,2). Those in congregate settings such as licensed child care or preschool also are at greater risk for influenza exposure and have the potential to expose many more persons than those outside of such settings. Similar to other vaccine-preventable diseases that are spread by respiratory droplets and that affect children, achieving high vaccination rates against influenza in child care settings not only protects those who are vaccinated, but also reduces transmission of influenza within the setting and to the associated outside community (8,9).

Although almost every state has adopted licensed child care requirements for other routinely recommended childhood vaccinations, only two have adopted a requirement for influenza vaccination (6). The reasons behind this discrepancy have not been studied. However, it might be, in part, because influenza vaccine is not widely available until well after licensed child care begins in August or September, when requirements are enforced. In addition, surveying of licensed child care centers and preschools for compliance usually takes place in October, when influenza vaccination often is just beginning. Thus, additional efforts are required to monitor and enforce compliance of entry requirements. In addition, because influenza vaccination has been available for decades but never required, it might be more difficult to convince parents and guardians of its necessity.

What is already known on this topic?

Preschool-aged children are at increased risk for severe influenza-related illness and are a major source of influenza transmission within communities. Only two states and recently, New York City, require influenza vaccination for child care attendance. In September 2010, Connecticut became the second U.S. state to implement regulations requiring that all children aged 6–59 months attending a licensed child care program receive at least 1 dose of influenza vaccine each year.

What is added by this report?

After implementation in September 2010 of required influenza vaccination for children in licensed child care programs, vaccination coverage among children aged 6–59 months in Connecticut increased from 67.8% during the 2009–10 influenza season to 84.1% in 2012–13, and the influenza-associated hospitalization rate in 2012–13 among children aged ≤4 years, compared with the 2007–08 season, decreased by 12%, the largest percentage decrease among the 11 Emerging Infections Program sites. In addition, the ratio of the influenza hospitalization rate among children aged ≤4 years to the overall (i.e., all ages) influenza hospitalization rate was lower in Connecticut (0.53) than in any of the other 10 sites.

What are the implications for public health practice?

Requiring vaccination against influenza for licensed child care attendance appears feasible and might help reduce the number of cases of serious illness from influenza among children aged ≤4 years.

Several of these concerns were encountered in conducting this study in Connecticut. Conducting an influenza-specific survey in the winter or spring after the requirement went into effect required more resources and took 2 years to arrange. The actual statewide compliance during the first two influenza seasons (2010–11 and 2011–12) after the requirement went into effect is unknown. Without timely surveys, compliance might be less with influenza than with other vaccines, and also might wane over time. In addition, religious and medical exemptions from influenza vaccination were observed to be higher than for any other required vaccine. Nonetheless, following implementation of the requirement in Connecticut in September 2010, vaccination rates among all children aged ≤4 years by 2012–13 appeared to have increased more than expected compared with national rates, and the incidence of hospitalization with influenza among children aged ≤4 years decreased more than in other states that conducted similar surveillance.

The findings in this report are subject to at least four limitations. First, this was an ecologic analysis comparing trends in influenza hospitalizations and vaccination coverage and must be interpreted as such. Other factors might have contributed to the relative decrease in hospitalizations of young children in Connecticut. Second, the results of the child care survey of vaccination coverage for the 2012–13 influenza season do not include family day care homes, which also are covered by the influenza vaccination requirement and account for approximately 22% of all children in licensed child care in Connecticut, nor do they include preschools, which also are covered by the requirement. Third, the rates and dynamics of influenza circulation in each EIP site are different, and changes over time in one site compared with others must be interpreted with caution. Finally, EIP hospitalization rates include those for children aged <6 months although these children are not yet eligible for influenza vaccination.

Vaccination against influenza has gradually evolved from an elective annual event for selected groups at high risk for influenza complications to a recommended annual event for all persons aged ≥6 months (10). In congregate settings in which persons are at greater risk for both exposure and complications, such as child care programs and health-care settings, the current recommendations call for most persons to be vaccinated. The Connecticut study might be helpful to public health agencies elsewhere considering requiring influenza vaccination of children in licensed child care programs and preschools.

## Figures and Tables

**FIGURE:**
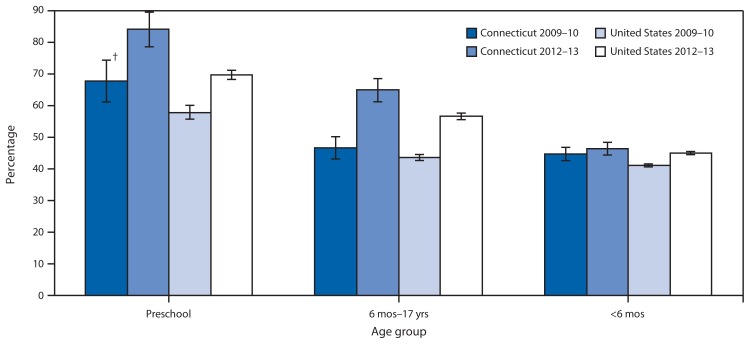
Seasonal influenza vaccination coverage, by age group — Connecticut and United States overall, 2009–10* and 2012–13 * Vaccination coverage for influenza A (H1N1)pdm09 was not included. ^†^ 95% confidence interval.

**TABLE 1:** Percentage change in the influenza-associated hospitalization incidence rate per 100,000 children aged ≤4 years — 11 Emerging Infections Program (EIP) sites, 2007–08 and 2012–13 influenza seasons

EIP site	2007–08 season		2012–13 season		(%) change from 2007–08 to 2012–13
	
	No. of cases	Rate per 100,000	No. of cases	Rate per 100,000	
California	72	36.2	82	40.8	(13)
Colorado	134	76.9	151	85.9	(12)
Connecticut	29	58.6	24	51.5*	(−12)
Georgia	52	18.9	145	53.6	(184)
Maryland	143	87.0	136	81.9	(−6)
Minnesota	91	46.9	148	76.0	(62)
New Mexico	35	45.4	73	82.0	(81)
New York – Albany	4	7.2	26	47.8	(564)
New York – Rochester	26	40.4	45	70.1	(74)
Oregon	19	18.4	21	19.7	(7)
Tennessee	34	33.8	42	40.3	(19)
**EIP sites overall**	**639**	**43.8**	**898**	**60.6**	**(38)**

* This incidence rate is different from the rate for Connecticut displayed in CDC’s FluView at http://gis.cdc.gov/grasp/fluview/fluhosprates.html. The rate in this table is based on New Haven County only, whereas the FluView catchment area for Connecticut expanded from one county in 2007–08 to three counties in 2012–13. During the 2012–13 influenza season, New Haven County had 24 cases and 46,626 children aged ≤4 years, based on the annual county population estimate used in FluView.

**TABLE 2:** Ratio of influenza-associated hospitalization incidence rate per 100,000 in children aged ≤4 years to the incidence rate for the population overall — 11 Emerging Infections Program (EIP) sites, 2007–08 and 2012–13 influenza seasons

EIP Site	2007–08 season		Rate ratio ≤4 yrs/overall	2012–13 season		Rate ratio ≤4 yrs/overall
	
	≤4 yrs rate	Overall rate		≤4 yrs rate	Overall rate	
California	36.2	10.3	3.30	40.8	34.5	1.18
Colorado	76.9	26.4	2.91	86.5	37.3	2.32
Connecticut	58.6	49.6	1.18	51.5*	96.5*	0.53
Georgia	18.9	9.5	1.99	53.6	31.1	1.72
Maryland	87.0	26.9	3.23	81.9	50.1	1.63
Minnesota	46.9	19.3	2.43	76.0	51.8	1.47
New Mexico	45.4	10.0	4.54	82.0	29.0	2.83
New York - Albany	7.2	8.8	0.82	47.8	43.8	1.09
New York – Rochester	40.4	37.3	1.08	70.1	95.7	0.73
Oregon	18.4	9.3	1.98	19.7	29.7	0.66
Tennessee	33.8	16.2	2.09	40.3	28.6	1.41
**EIP sites overall**	**43.8**	**18.3**	**2.39**	**60.6**	**43.2**	**1.40**

* These incidence rates are different from the rates for Connecticut displayed in CDC’s FluView at http://gis.cdc.gov/grasp/fluview/fluhosprates.html. The rates in this table are based on New Haven County only, whereas the FluView catchment area for Connecticut expanded from one county in 2007–08 to three counties in 2012–13. During the 2012–13 influenza season, New Haven County had 24 cases and 46,626 children aged ≤4 years, and overall had 833 cases and a total population of 862,813, based on the annual county population estimates used in FluView.

## Footnotes

* Additional information available at http://gis.cdc.gov/grasp/fluview/fluhosprates.html.

† Vaccination coverage for influenza A (H1N1)pdm09 was not included.
