# Errata

**Published:** 2014-03-14

## Vol. 63, No. 9

In the report, “Impact of Requiring Influenza Vaccination for Children in Licensed Child Care or Preschool Programs — Connecticut, 2012–13 Influenza Season,” on page 183, in the Figure, the groups of vertical bars were labeled incorrectly, and the legend was difficult to follow. The corrected Figure is as follows.

## Figures and Tables

**FIGURE:**
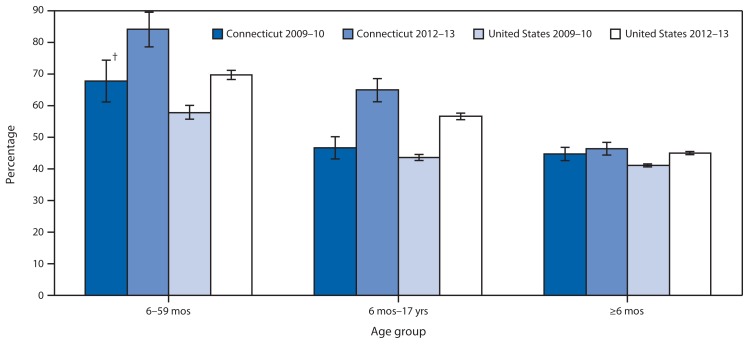
Seasonal influenza vaccination coverage, by age group — Connecticut and United States overall, 2009–10* and 2012–13 * Vaccination coverage for influenza A (H1N1)pdm09 was not included. ^†^ 95% confidence interval.

